# Visual estimates of blood loss by medical laypeople: Effects of blood loss volume, victim gender, and perspective

**DOI:** 10.1371/journal.pone.0242096

**Published:** 2020-11-12

**Authors:** Rachel Phillips, Marc Friberg, Mattias Lantz Cronqvist, Carl-Oscar Jonson, Erik Prytz

**Affiliations:** 1 Department of Psychology, Old Dominion University, Norfolk, VA, United States of America; 2 Department of Computer and Information Science, Linköping University, Linköping, Sweden; 3 Center for Disaster Medicine and Traumatology, Department of Biomedical and Clinical Sciences, Linköping University, Linköping, Sweden; University Hospital Zurich, SWITZERLAND

## Abstract

A severe hemorrhage can result in death within minutes, before professional first responders have time to arrive. Thus, intervention by bystanders, who may lack medical training, may be necessary to save a victim’s life in situations with bleeding injuries. Proper intervention requires that bystanders accurately assess the severity of the injury and respond appropriately. As many bystanders lack tools and training, they are limited in terms of the information they can use in their evaluative process. In hemorrhage situations, visible blood loss may serve as a dominant cue to action. Therefore, understanding how medically untrained bystanders (i.e., laypeople) perceive hemorrhage is important. The purpose of the current study was to investigate the ability of laypeople to visually assess blood loss and to examine factors that may impact accuracy and the classification of injury severity. A total of 125 laypeople watched 78 short videos each of individuals experiencing a hemorrhage. Victim gender, volume of blood lost, and camera perspective were systematically manipulated in the videos. The results revealed that laypeople overestimated small volumes of blood loss (from 50 to 200 ml), and underestimated larger volumes (from 400 to 1900 ml). Larger volumes of blood loss were associated with larger estimation errors. Further, blood loss was underestimated more for female victims than male victims and their hemorrhages were less likely to be classified as life-threatening. These results have implications for training and intervention design.

## Introduction

Trauma resulting in life-threatening bleeding can occur for a variety of reasons including accidents, assaults, mass casualty events, surgery, childbirth, armed conflict, and intentional self-harm. Depending on the location and severity of the injury, there may be as little as three minutes to stop the bleeding before the circulatory system irreversibly fails [1]. In many instances, life-threatening bleeding occurs outside of medical facilities and away from trained medical personnel. However, as recorded by emergency medical services, bystanders were present or arrived within minutes in more than half of the cases studied [2–4]. Bystanders who take action are referred to as immediate responders, and previous studies show that immediate responder first aid intervention rates range from approximately 11% [5] to 75% [6]. Thus, bystanders can play a pivotal role as immediate responders in the clinical outcome and survivability of traumatic bleeding injuries, provided they choose to assist and correctly initiate the necessary interventions.

In traumatic bleeding injuries, fast and accurate hemorrhage control may be the difference between life and death [7]. The amount and rate of blood loss, the location of the injury, and characteristics of the situation should all factor into the clinical decision process for appropriate interventions [8–10]. In a blood-loss related emergency, the immediate responders must determine if the injury is life-threatening and which hemorrhage control technique to use (e.g., direct pressure, a tourniquet, or wound packing combined with direct pressure; [11]). First responders and health care providers may also estimate the severity of bleeding and the amount of blood lost to determine the appropriate treatment actions [12–15]. As correct hemorrhage control depends, at least partially, on accurate subjective evaluation of the injury, it is particularly important to understand the factors that may influence injury severity estimates and related first aid decisions.

In a pre-hospital scenario, blood loss and injury severity estimates made by emergency personnel incorporate vital parameters such as heart rate and respiratory rate in addition to visual cues. However, medical novices (i.e., laypeople), such as most immediate responders, are unlikely to have the knowledge, skills, or equipment to incorporate vital parameters in their decision-making process. As a result, estimations of blood loss made by immediate responders are likely to rely heavily on visual examination of the injury and blood present at the scene. Unfortunately, visual estimation of blood loss tends to be inaccurate [12, 16–20]. People often overestimate small volumes and underestimate large ones [13, 21–26]. This tendency appears to occur regardless of level of medical expertise [27, 28], though people with less training or experience may be more inaccurate [29–32]. Overestimation of blood loss can lead to intervention decisions that expose individuals to unnecessary risks or it can reduce the availability of finite resources for other people. Underestimation of blood loss, on the other hand, can have potentially fatal consequences or lead to unnecessary medical complications.

Although previous research has shown that visual estimation of blood loss tends to be inaccurate, it is not clear how victim gender influences blood loss estimation accuracy. Victim gender may influence the likelihood of receiving assistance or the type of assistance received [5, 33, 34]. However, to date, there does not appear to be research examining the effects of victim gender on blood loss estimation accuracy, as shown by a recent literature review [35]. As some studies suggest gender differences in prehospital care [36] and the likelihood of being transferred to a level-1 trauma center when not medically necessary [37], it seems likely that victim gender will influence perceptions of injury severity and related first aid decisions.

Previous research on blood loss estimation has focused heavily on medical professionals or individuals with medical training. Additionally, much of this research has not systematically controlled for potential confounding variables such as the surface on which the blood was present, viewing angle, gender of the victim, viewing order, et cetera. Further, although different blood loss amounts have been used across a variety of studies, they have not necessarily been systematically varied to create a high-resolution mapping of estimation accuracy. Thus, one aim of the current study was to address these issues and establish a detailed baseline of blood loss estimations made by untrained laypeople in a controlled experiment. Another goal of the current research was to examine factors that influence blood loss estimation accuracy and perceptions of injury severity by potential immediate responders. Based on previous studies, we hypothesized that participants would 1) overestimate smaller volumes of blood and 2) underestimate larger volumes of blood. We also hypothesized that 3) the size of blood loss estimation errors would be impacted by blood loss volume and victim gender. In order to initiate an intervention, one must first classify the injury as serious. Thus, we examined participant classification of blood loss into two key categories, life-threatening or not life-threatening. We hypothesized that participants would be more likely to classify bleeding as life-threatening for 4a) higher volumes and for 4b) males versus females. Finally, as the speed of receiving an intervention can impact the survivability of the injury in cases of severe trauma, we were also interested in how quickly participants were able to make these classifications. We hypothesized that classification of blood loss would be faster for 5a) larger volumes, and for 5b) males compared to females.

## Method

The hypotheses were tested using a controlled experiment. Participants viewed 78 5-second video clips showing a male or female victim bleeding at different rates with different volumes of blood loss from different viewing perspectives (top-down or front). The perspective was a between-groups variable whereas victim gender and blood loss volume were within-groups variables.

### Participants

Students from a large university in the south east United States were recruited using an online participant management software system (SONA). A total of 81 participants were recruited into the top-view group. Of these, 24 were excluded from data analysis due to having prior healthcare experience or stop the bleed training. The remaining 57 participants (9 male, 48 female) ranged in age from 18–42 (*M* = 20.3, *SD* = 4.6). Further, a total of 94 participants were recruited into the front-view group. Results from 26 were excluded due to prior health care experience or stop the bleed training. The 68 participants (12 male, 56 female) included in the analyses ranged in age from 18–59 (*M* = 19.9, *SD* = 5.2). Participants received 1.5 research credits to use toward extra credit or class required research experience credits. The study was approved by the university institutional review board and all ethical guidelines from the American Psychology Association were followed.

### Materials

**Fig 1:**
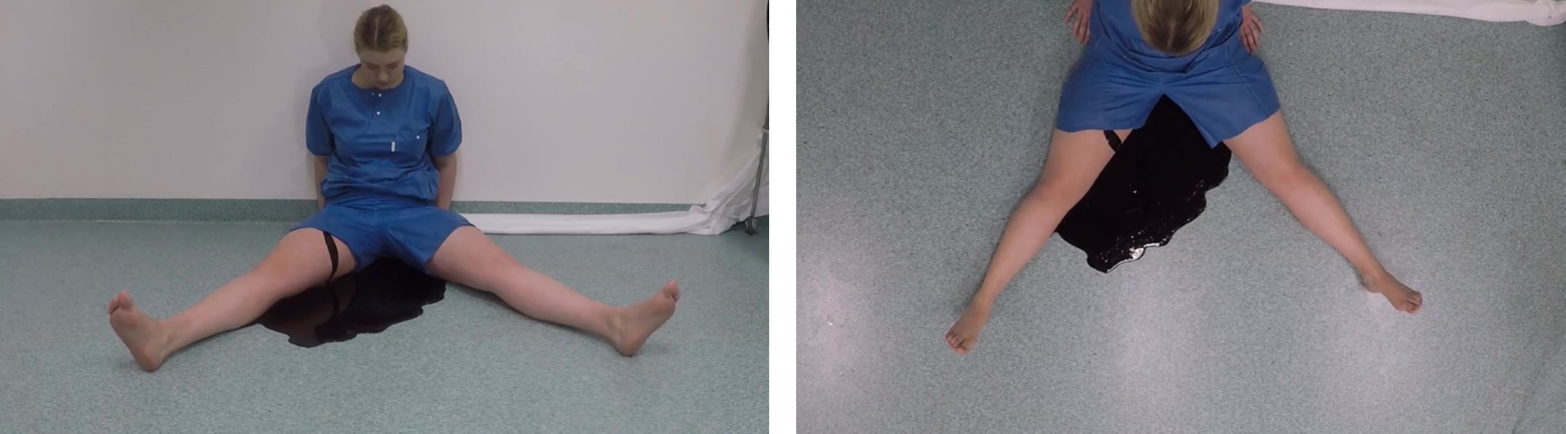
Example stimuli. Images showing the female actor from the front (left) and top (right) perspectives. The blood loss in this figure does not correspond to a specific volume used in the experiment. The individual shown in this figure has given written informed consent (as outlined in the PLOS ONE consent form) to publish these images.

To generate the stimuli for the experiments, videos were created to manipulate three variables: victim sex, blood volume, and the rate of blood flow. These videos featured an actor, either a man or a woman (180 and 171 cm tall, respectively), sitting on the floor with an identical position against a wall, wearing blue hydrophobic scrubs, and bleeding from the right inner thigh. The videos were filmed from straight above (top view) and from straight ahead with a slight downward slant (front view), see Fig 1.

Bleeding was simulated by pumping porcine blood through a plastic tube placed circa 10 cm distal to the pelvic area in the approximate location of the femoral artery. Porcine blood was used to mimic the color and viscosity of human blood. To ensure the blood was visible, the scrub pants were cut to mid-thigh length, just long enough to cover the plastic tube (i.e., the simulated injury) while providing an unobstructed view of the blood loss. The rest of the tubing was hidden by a white fabric and the pump was placed out of camera view. There was also a piece of plastic tubing taped under the actor’s legs to prevent blood from flowing underneath the actor where it would be obscured from view.

For each actor, three videos were recorded using different flow rates: 80, 200 and 400 ml/min. The videos were recorded until the blood loss exceeded 2000 ml or 25 minutes had elapsed. The videos were then divided into 5-second clips. The clips were created in such a way that the amount of blood present on the floor at the end of the clip was equal regardless of flow rate. To incorporate a wide range of volumes, 13 blood volume conditions were created: 0, 50, 100, 150, 200, 300, 400, 500, 700, 900, 1100, 1500, and 1900 ml. With two actors (male and female), three flow rates (80, 200, and 400 ml/min) and 13 blood volumes, there were a total of 78 video clips included in the study. Although the effect of flow rate was not of direct interest in the current study, and would be difficult to perceive during such short video clips, it was included as a variable during stimuli creation in order to have more than one video per gender and blood loss amount.

For each clip, the participants were asked to indicate if the bleeding was life-threatening or not while viewing the video. They also completed a questionnaire after each clip where they estimated the amount of blood present in either ml or fluid ounces (their choice). The participants also completed a demographics questionnaire after they had viewed all videos, indicating age, sex, ethnicity, health care experience, and if they had previous stop the bleed training.

### Procedure

The lab consisted of four computers, two on each side of the room separated by dividers. Thus, if the session was full, four participants completed the study at once. Participants arrived at the lab at their selected day and time. They were greeted by the experimenter and asked to read and sign the informed consent provided at their station. The completed forms were collected, and participants were then asked to provide their SONA ID numbers to ensure they received credit for the study. Consent forms and SONA ID numbers were collected and stored separately to ensure that they were not linked to each other or to the subsequent experimental data. After this, participants were informed that the remainder of the experiment would be completed on the computer. They were told that they would be viewing videos of simulated blood loss and asked to answer questions. Finally, they were asked to refrain from eating or drinking and to turn off and put away all electronic devices. After establishing that everyone understood, the experimenter instructed participants to click start and read the experimental instructions provided on the screen before beginning the experiment.

The experiment itself was completed using SABLE (System for Acquiring Blood Loss Estimates). SABLE was developed to automate the experimental process and store the data on a secure server. Participants were asked to click “Start” after reading the instructions provided by SABLE to begin the experiment. The video clips were shown in random order for each participant and were only visible for the 5-second duration of the clip. While the clip was visible, participants were asked to indicate if the bleeding was “life-threatening” or “not life-threatening” by clicking “A” or “D” on the keyboard. After the clip disappeared, participants completed the post-clip questionnaire using mouse and keyboard entry. This repeated for all 78 videos and then participants completed the demographic questionnaire. Finally, participants received a short debrief through SABLE that included a video clip of the female actor holding a sign saying “Thank you” to help offset any negative emotional effects of viewing the blood loss videos and to reinforce the understanding that the clips were simulations. The experimenter then read a debrief reminding participants that the videos were simulations, thanking them for their participation, and providing them with experimenter contact information if they had any questions. The experiment took between 40 and 55 minutes from arrival to departure.

### Analyses

The independent variables of interest included victim gender (male or female), the amount of blood lost (volume), and the perspective (top-view: looking down from above, front-view: looking straight at the scene). Volume and gender were within-subjects variables, perspective was a between-subjects variable. Dependent variables were volume estimations, estimation error, classification of life-threatening or not, and classification response time.

The data were checked for outliers and input errors. Particular care was taken with the volume estimations, as the participants could enter any number in either milliliters or fluid ounces. It was noted that some participants frequently used the wrong input box, e.g. provided an estimate in fluid ounces but entered the estimate in the ml input box. Therefore, all estimates from participants that showed inconsistency in the metric used, defined as providing less than 95% of their estimates using the same unit, were excluded. This resulted in the exclusion of estimates from five participants (8.77%) in the top-view condition and five participants (7.35%) in the front-view condition. The remaining data were screened for outliers, defined as data points more than three standard deviations from the mean. For the volume estimation variable, a total of 161 out of 4056 datapoints (4.1%) were removed from the data set for the top view, and 267 out of 4914 (5.7%) from the front view data set. Finally, one outlier, out of 4219 data points, was removed from the response time variable for the front view data set. The volume estimates, response times, and classifications were collapsed across flow rate to produce 26 answers (one per victim gender per 13 volumes) per participant. The volume of zero ml (control; no blood present) was excluded from the analyses. Data can be accessed through a repository [38].

A 12 (Volume) by 2 (Perspective) repeated measures ANOVA was conducted on the volume estimates to test the hypothesis that small volumes would be overestimated and large volumes underestimated. For the estimation error, response time, and classification variables, separate 12 (Volume) by 2 (Perspective) by 2 (Gender) repeated measures ANOVAs were conducted to test the other hypotheses. Lower-bound corrections were used for analyses that showed a significant Mauchly’s test of sphericity and the corrected F-string is reported for these. Data were analyzed using SPSS 25.

## Results

### Volume estimates

The 12 (Volume) by 2 (Perspective) repeated measures ANOVA for volume estimation showed a significant main effect of volume, corrected *F*(11, 109) = 147.095, *p* < .001, partial η^2^ = .574, but no effect of perspective, *F*(1, 109) = 1.002, *p* = .319, partial η^2^ = .009, or interaction, corrected *F*(11, 109) = 1.851, *p* = .176, partial η^2^ = .017. The effect of volume was best explained by a linear contrast, *p* < .001, partial η^2^ = .608, indicating that the estimates increased as the actual blood loss volume increased. Finally, 95% confidence intervals (CIs) were calculated for each of the 12 volumes (see Table 1).

**Table 1:** Means and 95% confidence intervals for the different volumes of blood loss.

Volume	Mean	SE	95% CI
50	106.1	7.1	[92.1, 120.1]
100	182.3	12.7	[157.0, 207.5]
150	231.0	17.1	[197.1, 264.9]
200	257.5	17.8	[222.2, 292.8]
300	294.1	19.8	[254.9, 333.2]
400	332.8	21.9	[289.3, 376.2]
500	390.0	27.3	[335.9, 444.2]
700	460.9	31.7	[398.2, 523.7]
900	549.1	38.6	[472.6, 625.6]
1100	659.9	47.7	[565.4, 754.4]
1500	836.5	62.2	[713.2, 959.8]
1900	947.3	69.2	[810.1, 1084.5]

**Fig 2:**
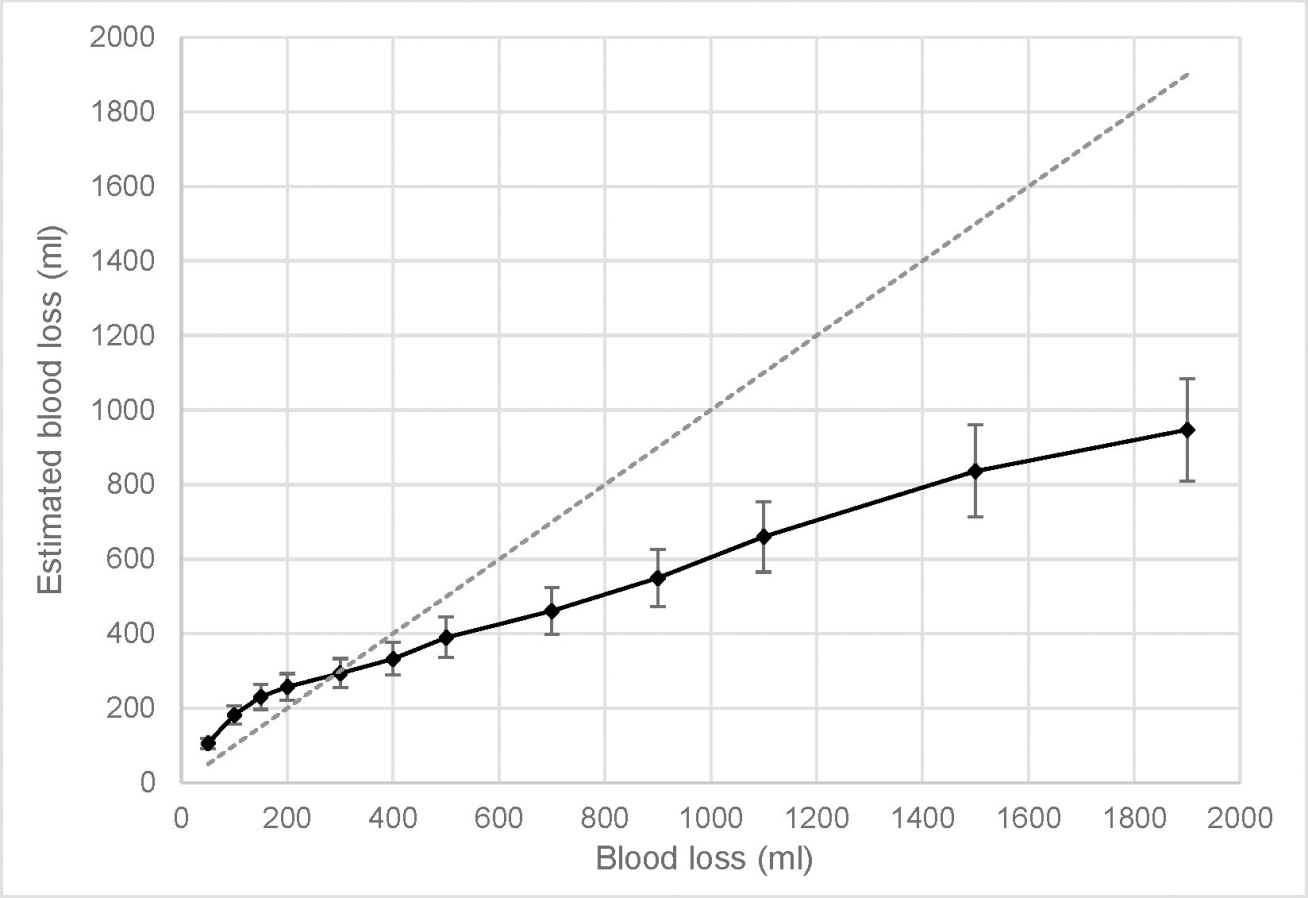
Estimated blood loss over actual blood loss. The black line shows mean estimates, with 95% CI as error bars. The dashed gray line indicates an accurate assessment.

The confidence intervals showed that blood loss in the lower ranges (50 to 200 ml) were overestimated, as the confidence interval exceeded the true volume. For 300 ml, the 95% CIs overlapped the true amount whereas volumes from 400 to 1900 ml were underestimated. This can also be seen in Fig 2.

### Estimation errors

**Fig 3:**
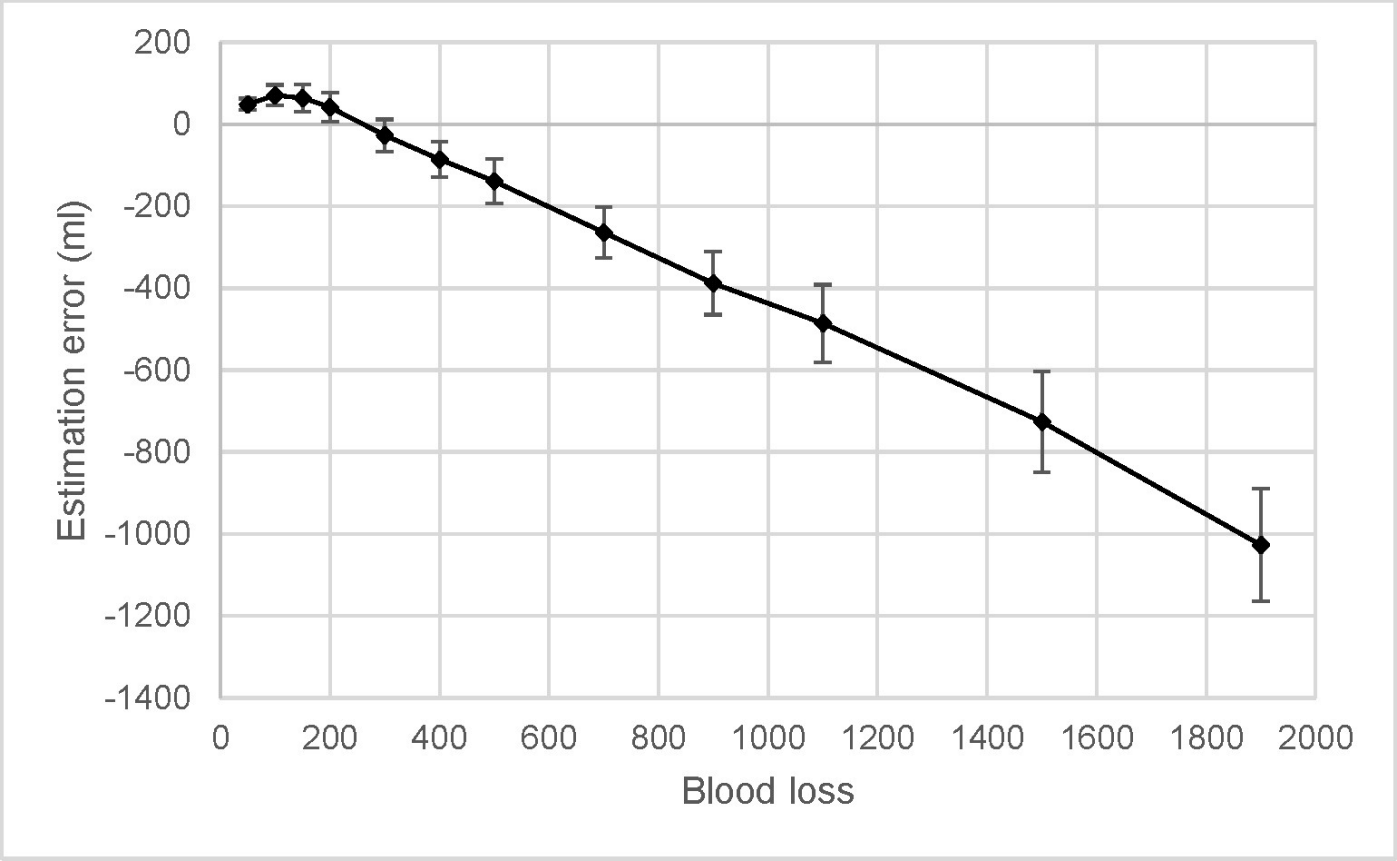
Estimation error over actual blood loss. The black line indicates the mean error, with 95% CIs as errors bars.

Estimation errors were calculated for each estimate made by each participant by subtracting the true amount from their estimate. A 2 (Gender) by 12 (Volume) by 2 (Perspective) repeated measures ANOVA showed a significant main effect of volume, corrected *F*(1, 103) = 297.151, *p* < .001, partial η^2^ = .743, and a main effect of gender, *F*(1, 103) = 76.337, *p* < .001, partial η^2^ = .426. The effect of volume was best explained by a quadratic trend, *p* < .001, partial η^2^ = .834. This can be seen as a slight positive increase in the estimation error up to about 100 to 150 ml, followed by a decrease indicating increased underestimation at higher volumes of blood loss, see Fig 3. The effect of gender was such that the blood loss was underestimated more for female victims (*M* = -264.87, *SE* = 27.55) than male victims (*M* = -221.81, *SE* = 29.53).

### Classification

**Fig 4:**
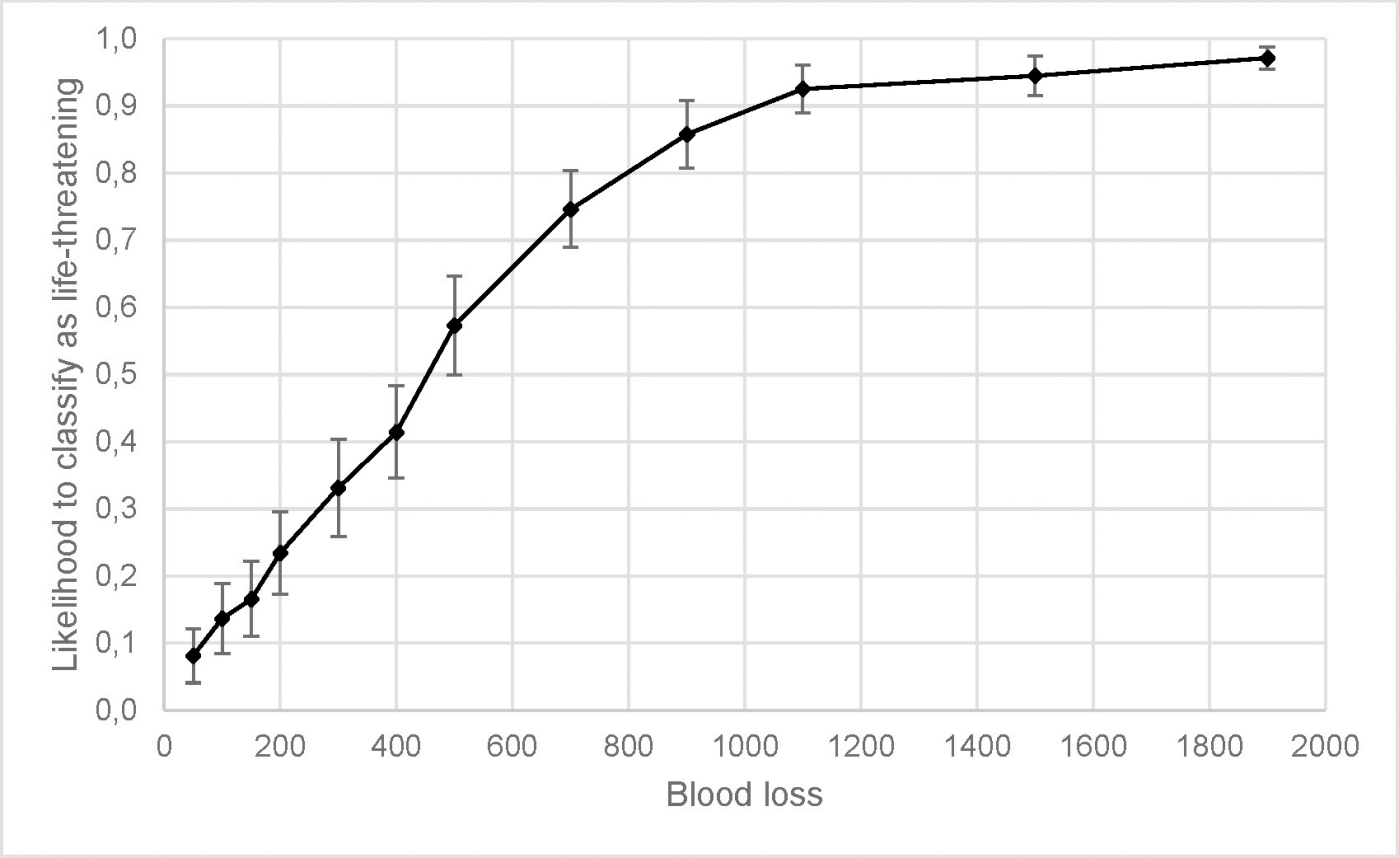
Mean likelihood that a video would be classified as showing a life-threatening bleeding over the blood loss volume. The errors bars are 95% CIs.

The 2 (Gender) by 12 (Volume) by 2 (Perspective) repeated measures ANOVA for classification as life-threatening or not life-threatening showed a significant main effect of volume, corrected *F*(1, 96) = 272.921, *p* < .001, partial η^2^ = .740, and gender, *F*(1, 96) = 54.379, *p* < .001, partial η^2^ = .362. The effect of volume was best explained by a linear contrast, *p* < .001, partial η^2^ = .912. Fig 4 shows the probability that a particular blood loss volume would be classified as showing a life-threatening bleeding. The effect of gender was such that videos with a female victim were less likely to be classified as showing a life-threatening bleeding (*M* = 0.498, *SE* = 0.019) as compared to videos with a male victim (*M* = 0.566, *SE* = 0.019).

There was also a significant interaction between gender and perspective for classification, *F*(1, 96) = 4.815, *p* = .031, partial η^2^ = .048. However, follow-up analyses failed to identify any significant differences, likely due to the small effect.

### Response time

**Fig 5:**
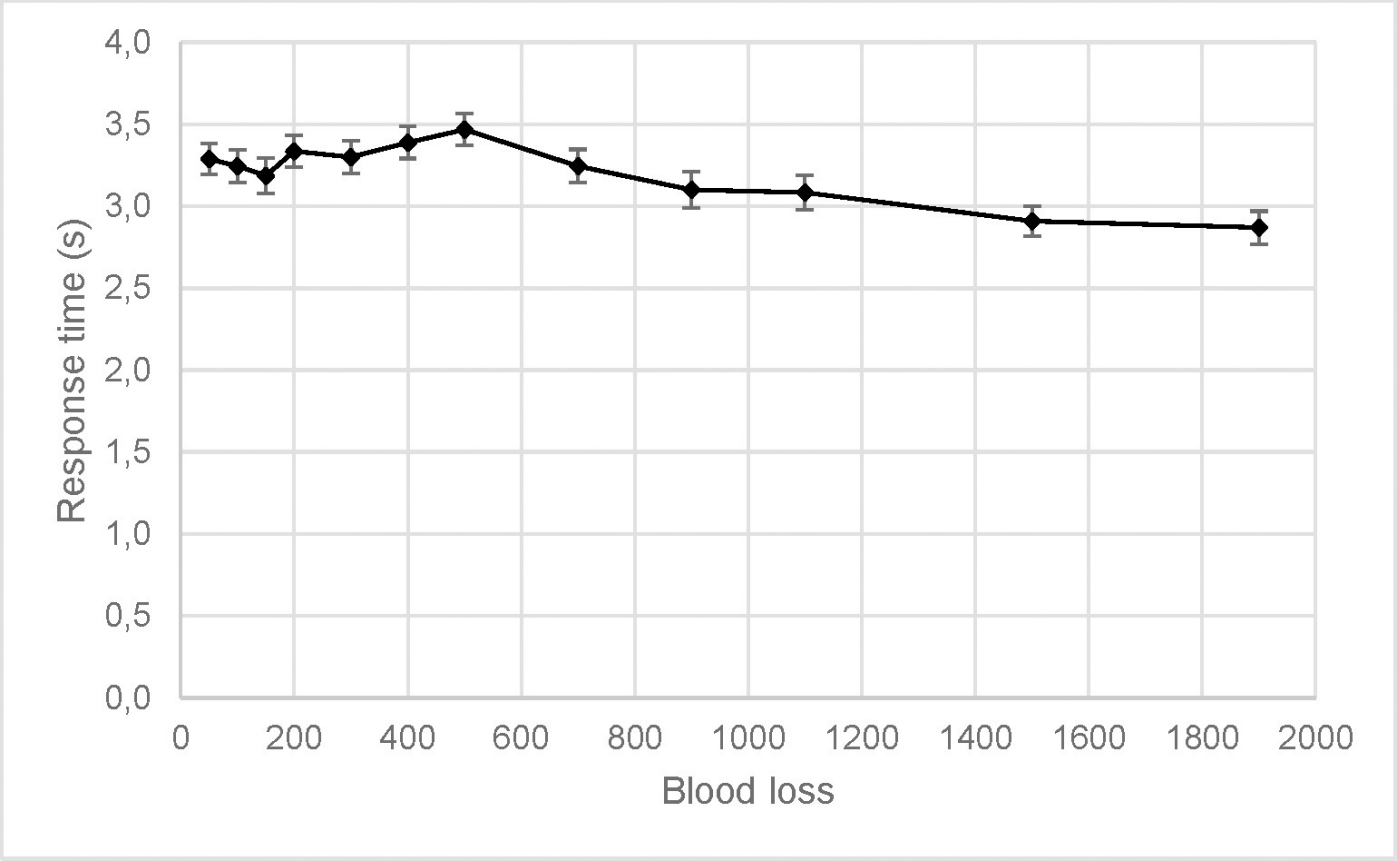
Response time over blood loss volume. The black line shows the mean response time, with 95% CI as error bars.

The response time for the participants’ initial classification of the bleeding as life-threatening or not was also analyzed using a 2 (Gender) by 12 (Volume) by 2 (Perspective) repeated measures ANOVA. The analysis showed a significant main effect of volume, corrected *F*(1, 96) = 25.965, *p* < .001, partial η^2^ = .213, and gender, *F*(1, 96) = 17.130, *p* < .001, partial η^2^ = .151. There was also a significant interaction effect between gender and perspective, *F*(1, 96) = 13.039, *p* < .001, partial η^2^ = .120. The interaction effect was such that the participants had faster response times to the male videos (*M* = 3.234, *SE* = 0.071) than female videos (*M* = 3.395, *SE* = 0.049) for the top perspective, *t*(47) = 3.291, *p* < .01, but there was no difference between male (*M* = 3.195, *SE* = 0.052) and female (*M* = 3.204, *SE* = 0.053) videos for the front perspective, *t*(62) = 0.389, *p* = .698. The main effect of volume was best explained by a quadratic contrast, *p* < .001, partial η^2^ = .409. As can be seen in Fig 5, the response time increased somewhat from 50 ml blood loss to 500 ml, only to decrease again as the blood loss increased.

## Discussion

Participants estimated the amount of blood loss and classified the injury as life-threatening or not for 78 5-second video clips. Volume estimation, estimation error, classification tendency, and response time were examined in relation to victim gender, blood volume, and video perspective. The hypotheses that small volumes of blood would be overestimated and large volumes underestimated were supported by the results. This replicates the general findings from previous research, e.g. [13, 21–26]. Additionally, as the current study included 12 distinct blood loss volumes, it allowed for a closer examination of over- and underestimation behavior. Based on the results, volumes up to 200 ml were overestimated, volumes larger than 400 ml were underestimated, and volumes of 300 ml were generally accurately estimated. Most participants estimated in fluid ounces and 10 fluid ounces corresponds to approximately 300 ml. Thus, the relative accuracy at 300 ml could be an artefact of the unit of measurement most participants used in combination with people’s tendency to estimate in round numbers. Future studies should examine this possibility.

We also hypothesized that the blood loss estimation errors would be affected by the volume of blood loss and the gender of the victim. These hypotheses were supported. Specifically, estimation errors first increased in a positive direction (greater overestimations) up to about 150 ml, and then increased in a negative direction (greater underestimations) from there. This again mirrors previous research with trained medical professionals [27, 28] and extends those findings to include novices. Further, blood loss was underestimated more for female victims than male victims, regardless of volume. Much of the previous research on this topic has focused on a single gender (such as obstetrics) or has not included gender as a variable [35]. However, for trauma cases this could have a significant impact on how bystanders choose to act and respond to a bleeding victim.

As hypothesized, the effect of gender is further reflected in the classification scores, as blood loss was less likely to be classified as life-threatening for videos with the female victim than videos with the male victim. This has potential to impact first aid decisions in response to traumatic injuries as injury severity may be underestimated more for females. As hypothesized, there was also an effect of the blood loss volume with higher volumes being more likely to be classified as life-threatening. For example, 90% of respondents classified blood loss over 1000ml as life-threatening whereas only 50% of respondents classified blood loss of 400–500 ml as life-threatening. Recommendations from the stop the bleed education consortium [7] suggest teaching laypeople to treat blood loss of 150 ml as life-threatening. However, in the current study only 17% of respondents classified 150 ml of blood loss as life-threatening. Though it may be tempting to teach potential immediate responders to treat greater than minimal bleeding as life-threatening, thus circumventing the problem of estimation inaccuracy, in mass casualty incidents this may result in a misappropriation of scarce resources such as tourniquets. Thus, training programs aimed at laypeople potentially need to include estimation training or simple estimation techniques (such as the MAR method; [39]) to facilitate proper first aid interventions for bleeding injuries.

We further hypothesized that classification of blood loss as life-threatening or not would be faster for larger volumes. This was partially supported. Response times increased somewhat from 50 ml blood loss to about 500 ml, and thereafter decreased as blood loss increased. It is plausible that higher volumes of blood loss, though underestimated, are easier to identify as life-threatening and thus are more quickly classified. On the other hand, moderate amounts of blood loss may be perceived as more ambiguous and, therefore, require more time to process. This is supported by the observed classification scores, where more than half of participants classified blood loss volumes greater than 400 ml as life-threatening. As the volume of blood present increased, the percentage of respondents classifying the blood loss as life-threatening increased and the classification response times decreased.

Related to the hypothesis that response times were faster for males compared to females, there was a main effect of gender, as well as an interaction effect between gender and perspective. In line with our hypothesis, response times were faster for males compared to females. As argued in the preceding paragraph, response time is likely tied to classification ambiguity. One potential explanation is related to differences in the estimated blood loss between genders. Specifically, blood loss volumes were perceived to be higher for the male victim compared to the female victim. The perception of decreased blood loss volume for the female victim may have increased the ambiguity of the situation and thus increased the amount of time required to make a classification. As the injury was also less likely to be classified as life-threatening for the female victim, it seems likely that there is some form of gender bias at work. Future research should investigate the mechanisms behind the bias and its impact on the decision making process in this context.

The interaction effect was such that participants had faster response times for males than females, but only for the top perspective. Visual perceptual cues such as morphology play a role in judgement and perception of sex and gender [40]. Some morphological features may become more apparent when viewed from a particular angle and thus may have more or less impact on decision processes depending on the viewer’s perspective. This topic is relevant in real-life scenarios and should be further explored.

The current research examined laypersons’ ability to visually estimate blood loss. Our findings mirror past research with trained medical professionals. However, future research should replicate the current findings with a professional population using the same methodology to examine the estimation accuracy and response patterns more in depth, particularly as related to gender. When examining medical professionals it would also be beneficial to examine the relationship between blood loss estimation errors and shock class classifications as these are often used in trauma education [41]. This could further be compared to the current baseline for laypeople to identify differences between the populations. Identifying clear differences between the populations would allow for a greater understanding of the effectiveness of current medical training practices for overcoming inherent perceptual biases. Effective practices could then be leveraged to enhance hemorrhage control training programs for laypersons. However, if the same perceptual biases occur between populations, this may indicate that these biases are robust and need to be accounted for in medical decision making and training programs.

Other possible areas for future research could include characteristics of the stimuli and how the stimuli are presented. In the current study, to control as many extraneous variables as possible, the clips contained the same male and female victim in identical clothing and positions with no context aside from the blood flowing from a concealed wound. Victim characteristics such as BMI, age, clothing, and ethnicity could potentially play a role in visual blood loss estimation as could the source of bleeding, characteristics of the injury, and the situation in which the bleeding occurs. These factors may impact not only blood loss estimation accuracy, but also response times and first aid related decisions. Finally, as experts and novices were found to view accident scenes differently [42], it seems likely that there may also be variations in where experts and novices focus when viewing a hemorrhaging victim. Therefore, incorporating eye-tracking may be beneficial.

Laypersons are potential immediate responders, and as such, it is crucial to understand how they perceive hemorrhage scenarios and which factors may influence these perceptions. This study examined how untrained laypersons visually estimated and categorized blood loss in an experimental setting. In the current study, volumes of 200 ml or less were overestimated whereas volumes of 400 ml or more were underestimated. The results also indicated that blood loss volume and gender influence the blood loss estimation errors, the classification of hemorrhage–if it is life-threatening or not–and the response times of the classifications. Specifically, higher volumes of blood loss were associated with larger estimation errors but were more likely to be classified quickly and considered life-threatening. Blood loss estimations and the likelihood of classifying bleeding as life-threatening tended to be higher for males than for females and, for one viewing perspective, response times were also faster for male victims. Thus, it appears that visual blood loss estimation and injury classification by laypeople are influenced by the amount of blood present, victim gender, and, to a lesser extent, viewing perspective. Future hemorrhage control training programs should be designed to address these pre-existing tendencies or biases to enhance learning outcomes.
